# Gabapentin as Add-On to Fentanyl and Midazolam in Patients Receiving Mechanical Ventilation: A Randomized, Blinded Study

**DOI:** 10.5152/TJAR.2022.21366

**Published:** 2022-04-01

**Authors:** Sara Salarian, Elham Memary, Farinaz Taheri, Bahador Bagheri

**Affiliations:** 1 Anesthesiology Research Center, Shahid Beheshti University of Medical Sciences, Tehran, Iran; 2 Cancer Research Center, Semnan University of Medical Sciences, Semnan, Iran; 3 Center for Molecular Cardiology, University of Zurich, Schlieren, Switzerland

**Keywords:** Fentanyl, gabapentin, ICU, mechanical ventilation, sedation

## Abstract

**Objective:**

Fentanyl and midazolam are popular drugs for sedation and analgesia in intensive care unit. Gabapentin has sedative and analgesic effects, as well. Our purpose was to study gabapentin addition to fentanyl and midazolam to reach the target sedation level in patients requiring mechanical ventilation.

**Methods:**

This was a randomized and double-blinded trial. Fifty patients receiving mechanical ventilation and aged from 18 to 70 years were randomized 1 : 1 to 300 mg gabapentin q8hr or placebo. The initial infusion rates of fentanyl and midazolam were 1-2 µg kg^-1^ h^-1^ and 0.06-0.2 mg kg^-1^ h^-1^, respectively, in both groups. Treatments continued prior to weaning. Ramsay sedation scale score (2-3) and behavioral pain scale score (≤4) were used for the evaluation of sedation and analgesia levels, respectively.

**Results:**

A total of 43 patients were studied. Both treatment modalities reached the target sedation and analgesia levels. In the intervention group, there were significant reductions in daily consumption of fentanyl and midazolam (*P* < .01). Duration of ventilation was shorter in the intervention group (4.1 ± 1.7 days vs 5.1 ± 1.3 days, *P* > .05). There was no significant difference in intensive care hospitalization, although it was shorter in the intervention group (201 ± 24 hours vs 224 ± 19 hours, *P* > .05).

**Conclusions:**

This trail showed that both treatment modalities could reach target sedation and analgesia levels without significant differences. Add-on therapy with gabapentin could reduce the total dose of fentanyl and midazolam.

**Main Points**

- Gabapentin is useful in patients requiring mechanical ventilation.
- Gabapentin can be added to fentanyl and midazolam.

Gabapentin can reduce total requirement for fentanyl and midazolam.

## Introduction

Adequate level of sedation is a cornerstone in intensive care unit (ICU) patients who require mechanical ventilation.^1^ Cardiovascular and respiratory depression, tolerance, dependence, delirium, and unplanned extubation are current issues due to uncontrolled level of sedation. Fentanyl and midazolam are widely used for the management of patients during ventilation. Fentanyl is a synthetic opioid with significant analgesic and sedative effects. Midazolam is a benzodiazepine that augments γ-aminobutyric acid (GABA) effects and is usually associated with deep sedative and hypnotic effects. Of note, tolerance, withdrawal, and respiratory depression are important untoward effects of opioids and benzodiazepines.^2-4^ At present, standard practice for sedation and analgesia in critical patients is a subject of debate. In recent years, clonidine, dexmedetomidine, and gabapentin have been considered as helpful drugs in ICU.^5,6^ Gabapentin is an antiepileptic drug with established sedative and analgesic effects. It is structurally related to GABA but has no GABAergic actions. Gabapentin is supposed to have effects on dorsal root ganglia and spinal cord. Its calcium channel blocking effects may explain the analgesic effects of gabapentin. In addition, gabapentin carries an acceptable safety profile without serious concerns due to side effects.^7,8^ There is a limited amount of data to conclude whether gabapentin combination with benzodiazepines and opioid is associated with positive effects in critical settings. This trial was designed to study whether gabapentin addition to fentanyl and midazolam can achieve target sedation in patients undergoing ventilation.

## Methods

### Study Design

This randomized, double-blinded, and placebo-controlled study was conducted in a general hospital in Tehran, Iran. The local ethics committee has approved the study, and written informed consent was taken prior to trial participation. The study was performed according to the World Medical Association Declaration of Helsinki, and it was registered in Iranian Registry of Clinical Trials IRCT20190202042588N1. Fifty patients aged from 18 to 70 years receiving mechanical ventilation for more than 3 days were included. Sealed envelopes were used for allocation concealment, and random number table was used for randomization of patients. Average length of ICU stay was 7 days. Patients were randomly divided 1 : 1 into 2 groups. In the control group, patients were given placebo (Amin Daru, Isfahan, Iran), and in the intervention group, 300 mg gabapentin tablet (Tolid Daru Pharmaceuticals, Tehran, Iran) was dissolved in water and given every 8 hours via nasogastric tube. The initial infusion rates of fentanyl and midazolam were 1-2 μg kg^-1^ h^-1^ (Caspian Pharmaceuticals, Iran) and 0.06-0.2 mg kg^-1^ h^-1^ (Caspian Pharmaceuticals, Iran), respectively, in both groups.^9,10^ According to our protocols, adjustments in infusion rates were made to reach Ramsay sedation score (RSS) of 2-3 every 10 min.^11^ Participants were excluded if they had liver disorders (liver enzyme abnormalities >3 upper limit of normal), severe renal failure (high creatinine level according to age), severe neurological disorders (low level of consciousness, neuromuscular disorders), head trauma, brain hemorrhage, hemodynamic instability (low systolic blood pressure), receiving rescue therapies, inability to receive drugs enterally, and history of hypersensitivity. Study subjects were discontinued in case of these conditions: lost to follow-up and safety reasons. The study medications were titrated to maintain the target sedation level. Bolus doses of fentanyl and midazolam were used in case of inadequate sedation or analgesia. In addition, behavioral pain scale (BPS) score was used for evaluation of analgesia level.^12^ Target analgesia level was ≤ 4. The study medications continued prior to a successful weaning. Spontaneous breathing trial (SBT) was used for weaning.^13^ Criteria for successful SBT were respiratory rate < 35 breaths min^-1^, heart rate < 140 min^-1^, arterial oxygen saturation > 90%, systolic blood pressure > 80 mm Hg, and absence of respiratory signs. Previously, we had used a rapid shallow breathing index for weaning.^14^ Patients were followed-up till ICU discharge.

### Efficacy and Safety Assessment

The primary end point was the average sedation score in both treatment groups. Average analgesia score, total dose of fentanyl and midazolam, length of ICU stay, and unwanted effects of medications including respiratory depression, hypotension (mean arterial pressure less than <20% of baseline), bradycardia, and delirium were the secondary end points. Patients were continuously monitored for sedation, analgesia, and safety of drugs.

### Data Analysis

To study differences in level of sedation, a total number of 50 patients were included for randomization according to the assumption of 10% dropout in number of the study patients with a significance level of .05 and power of 80%. Shapiro–Wilk test was used to test normal distribution of data. Student’s *t* test was used for mean comparisons. We used *χ* ^2^ and Fisher’s exact test to study the associations between variables. *P* < .05 was statistically significant. The Statistical Package for Social Sciences, version 19.0 software (SPSS Inc.; Chicago,IL, USA) was used for data analysis.

## Results

### Baseline Characteristics

**Figure 1.**
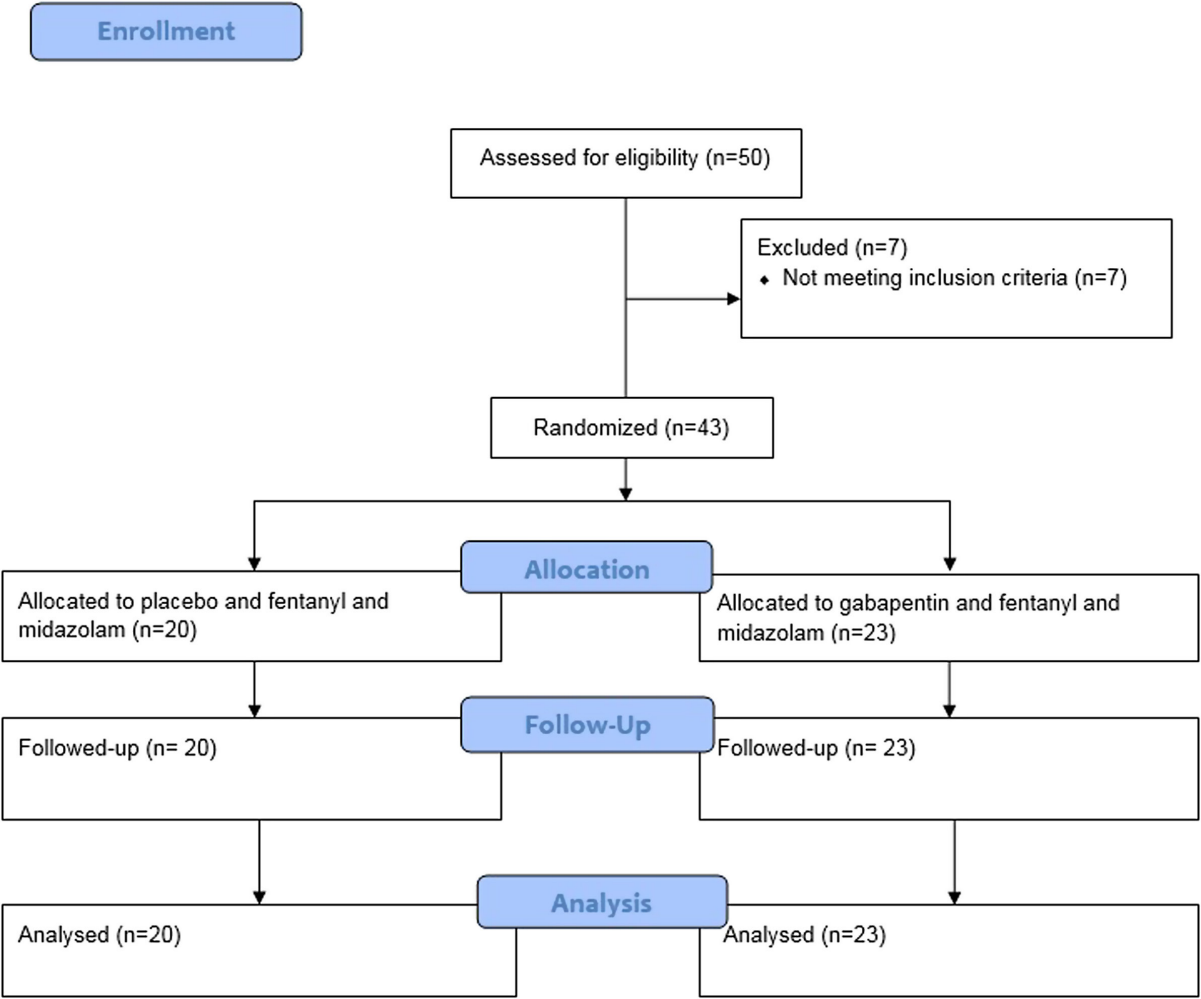
Consort diagram detailing study subjects.

Of the 50 study subjects who were included, 7 patients did not receive the treatments (4 met the exclusion criteria and 3 had mechanical ventilation for less than 3 days). No patient was lost to follow-up, and all 43 participants were observed over the course of ventilation support. Patients were investigated between September 2019 and March 2020. Table 1 shows the clinical characteristics of study participants, and there were no significant differences. The mean age was 55 years with an excess of females (53% vs 47%). Patients’ flow through is shown in Figure 1.

### Gabapentin Impact on Sedation and Analgesia

**Figure 2.**
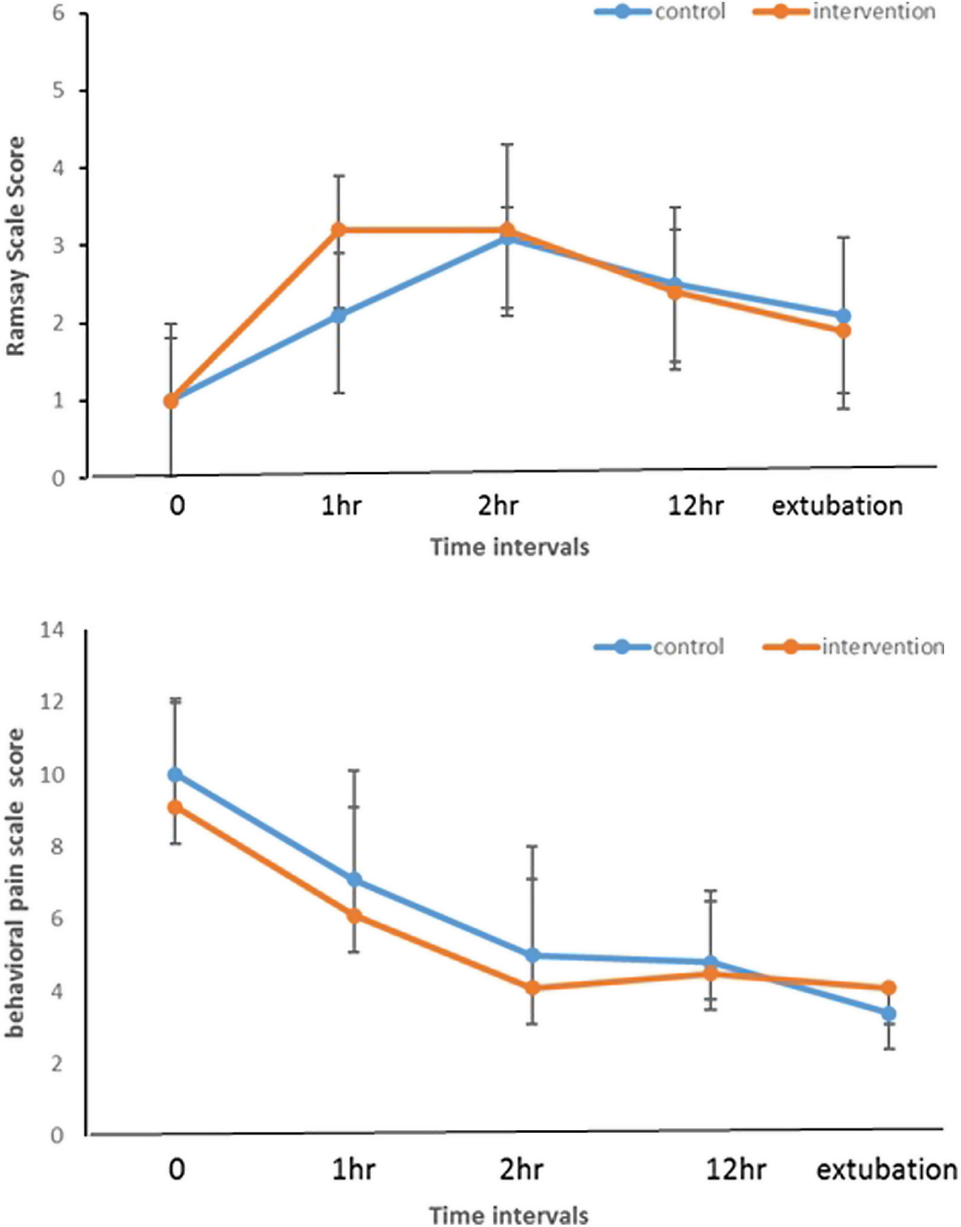
Comparison between 2 groups on the level of sedation (Ramsay scale score) and level of analgesia (behavioral pain scale score).

As indicated in Figure 2, target sedation level (RSS = 2-3) was reached in both study groups. There were no significant differences between 2 groups at different time intervals (*P* > .05). Ramsay sedation score was slightly higher in intervention group at 1 hour. Moreover, both treatment modalities could reach the target analgesia level (BPS ≤ 4). Differences were not statistically significant between 2 study groups. However, BPS was slightly lower in the intervention group at 1, 2, and 12 h.

### Gabapentin Impact on Fentanyl and Midazolam Requirement

Over the course of ventilation, fentanyl and midazolam doses were significantly lower in the intervention group as compared to control group (Table 2). Mean dose of fentanyl was 4.1 ± 1.5 μg kg^-1^ h^-1^ in the control group and 1.8 ± 0.1 μg kg h in the intervention group (*P* < .01). Moreover, mean daily dose of midazolam was 1.2 ± 0.1 mg kg^-1^ h^-1^ in the control group and 0.06 ± 0.04 mg kg^-1^ h^-1^ in the intervention group (*P* < .01). No patient received additional doses of fentanyl and midazolam in the intervention group, but 2 patients in the control group received an increased rate of fentanyl. In addition, no significant difference was noted in the heart rate and respiratory rate between 2 study groups (*P* < .5).

### Gabapentin Impact on Duration of Ventilation

As shown in Table 2, although the time on mechanical ventilation was shorter in the intervention group, differences were not significant; it was 5.1 ± 1.3 days in the control group and 4.1 ± 1.7 days in the intervention group (*P*  = .7).

### Gabapentin Impact on Length of Intensive Care Unit Stay

As presented in Table 2, ICU stay (time between arrival and ICU discharge) was a bit shorter in the intervention group. However, no significant difference in ICU stay was reported in both groups. It was 224 ± 19 hours in the control group and 201 ± 24 hours in the intervention group (*P*  = .6).

### Adverse Effects of Treatments

Patients in the control group experienced delirium more frequently (45% vs 17%, *P* < .05, Table 2). Besides, there were no significant differences in the frequency of hypotension, bradycardia, and respiratory depression between study patients. None of the included subjects died and no one was withdrawn due to severe adverse effects of treatments.

## Discussion

To our knowledge, this is the first trial that provided data about gabapentin effects in ventilated patients. Our result showed that the addition of gabapentin to fentanyl and midazolam could significantly reduce their daily requirements while reaching the desired level of sedation. In addition, time on the mechanical ventilation and ICU stay was shorter than the intervention group, but the differences were not statistically significant. Reduction in the amount of fentanyl and midazolam is important for safety importance, and it can affect rate of delirium, duration of ventilation, and may be hospital stay. It is well known that fentanyl and midazolam are associated with respiratory depression, dependence, and withdrawal symptoms.^2-4^ In general, any practice to achieve target sedation level by low doses of fentanyl and midazolam would be of interest in ICU. Our previous experience showed that clonidine can cause an acceptable level of sedation and can reduce total doses of fentanyl and midazolam in patients receiving mechanical ventilation.^15^ In addition, combination of fentanyl and midazolam has been resulted in a decrease in rate of unplanned extubation as compared to either fentanyl or midazolam alone.^16^ Current data about gabapentin effects in ICU are limited. Two randomized placebo-controlled trails by Pandey et al^17^ provided primary evidence for gabapentin use in ICU. In the first trial, it was shown that 7-day treatment with gabapentin (15 mg kg^-1^ day^-1^) was effective to relieve pain in 18 patients with Guillain-Barré Syndrome (GBS) who were admitted to ICU and required mechanical ventilation. Their other investigation proved that 300 mg gabapentin (q8hr) was associated with a faster onset of analgesia as compared with 100 mg carbamazepine (q8hr) in GBS patients requiring mechanical ventilation. Gabapentin could also decrease daily requirement for fentanyl.^18^ In both studies, gabapentin was considered a safe medication. Preanesthetic and preoperative effects of gabapentin have been subjects of research in recent years. The study by Hosokawa et al.^19^ demonstrated that 400 mg dose of gabapentin reduced the propofol target blood concentration for achieving sufficient level of sedation in 10 healthy volunteers. A work by Bharti^20^ reported that preoperative administration of gabapentin 600 mg significantly decreased propofol requirement. In addition, gabapentin was effective to reduce the demand for postoperative analgesics. Bharti et al’s^20^ study demonstrated no significant difference in fentanyl consumption between 2 groups of patients who received the preemptive dose of gabapentin. Unlike these 2 studies, we used a smaller dose: gabapentin 300 mg. It is noteworthy that several works have investigated the sedative and analgesic effects of gabapentin in pediatrics.^21-23^ They mostly emphasized on acceptable safety and efficacy of gabapentin. A wealth array of evidence considers gabapentin as an anti-seizure drug with sedative and analgesic properties, yet its mechanism of action remains to be understood. Gabapentin is able to modulate α2δ-1 and α2δ-2 auxiliary subunits of calcium channels and blocks excitatory neurotransmission via *N*-methyl-d-aspartate and a-amino methyl propionic acid receptors. Gabapentin may interrupt the recycling of calcium channels in the dorsal horn and hence the transmitter release is suppressed. Of note, gabapentin is able to inhibit excitatory synaptogenesis.^24,25^ By these mechanisms, gabapentin can interrupt epileptogenesis and nociception. However, it is not known with certainty that how gabapentin causes sedation. Current reports show that analgesic and sedative effects of gabapentin are achieved by doses of 300 to 1800 mg with various degrees of response. Gabapentin has generally an acceptable safety profile without significant drug interactions. Dizziness and drowsiness are the most frequently reported side effects. Of note, a dangerous warning exists about multiorgan hypersensitivity which is quite rare. Furthermore, some studies have reported that gabapentin may deteriorate the hemodynamic status of critical patients.^26,27^ However, we reported no patient with significant hemodynamic problems. Finally, it should be noted that current clinical practice guidelines for the prevention and management of pain, agitation/sedation, delirium, immobility, and sleep disruption in adult patients in ICU strongly recommends gabapentin with opioids for the management of neuropathic pain in critically ill patients.^28^

Our findings should be confirmed in more patients with longer time frames. Moreover, the effects of gabapentin on arousability, hospital stay, and post-intensive care syndrome can be addressed by future works.

## Conclusion

This trail showed that both treatment modalities could reach target sedation and analgesia levels without significant differences. Add-on therapy with gabapentin could reduce the total dose of fentanyl and midazolam.

### Declaration of Interests:

The authors have no conflict of interest to declare.

## Figures and Tables

**Table 1.** Characteristics of Patients at Baseline in 2 Study Groups

**Characteristics**	Control (n = 20)	Intervention (n = 23)	*P*
Age (years)	52.4 ± 8.3	57.7 ± 9.5	.5
Age (range)	20-65 years	19-68 years	.4
Female	11 (55)	12 (52)	.7
Body weight (kg)	78 ± 1.8	83 ± 7.9	.3
Admission diagnosis			
Pneumonia	6 (30)	9 (39)	.7
Sepsis	4 (20)	5 (21.7)	.8
Trauma	6 (30)	7 (30.4)	.6
Surgery	4 (20)	2 (8.6)	.7
Other	1 (5)	2 (8.6)	.4
Hemoglobin (g L^-1^)	102 ± 4.9	102 ± 4.2	.2
Total WBC count (10^9^ L^-1^)	7.3 ± 3.3	7.3 ± 2.9	.6
Platelets (10^9^ L^-1^)	213 ± 111	223 ± 109	.5
SBP (mm Hg)	133 ± 6.2	129 ± 3.1	.5
DBP (mm Hg)	60 ± 5.9	63 ± 6.4	.5
Respiratory rate (breaths min^-1^)	15 ± 1.1	17 ± 2.3	1.0
Heat rate (beats min^-1^)	73 ± 1.0	71 ± 1.1	.4

Data are shown as mean ± SD or number (%).

SD, standard deviation; WBC, white blood cells; SBP, systolic blood pressure; DBP, diastolic blood pressure.

**Table 2.** Comparison Between 2 Study Groups on Daily Doses of Midazolam and Fentanyl, Duration of Ventilation, ICU Stay, and Frequency of Side Effects

	Control (n = 20)	Intervention (n = 23)	*P*
Midazolam daily dose (mg kg^-1^ h^-1^)	1.2 ± 0.1	0.06 ± 0.04	< .01
Fentanyl daily dose (μg kg^-1^ h^-1^)	4.1 ± 1.5	1.8 ± 0.1	< .01
Duration of ventilation (day)	5.1 ± 1.3	4.1± 1.7	0.7
Length of ICU stay (h)	202 ± 19	197 ± 24	0.6
Delirium	9 (43)	4 (17.3)	0.03
Hypotension	3 (15)	1 (4.3)	0.5
Bradycardia	2 (10)	2 (8.6)	0.8

Data are shown in mean ± SD or number (%). SD, standard deviation; ICU, intensive care unit.

## References

[1] Chanques G Jaber S Barbotte E , et al. Impact of systematic evaluation of pain and agitation in an intensive care unit. Crit Care Med. 2006;34(6):1691 1699. 10.1097/01.CCM.0000218416.62457.56) 16625136

[2] Robinson BR Mueller EW Henson K Branson RD Barsoum S Tsuei BJ . An analgesia-delirium-sedation protocol for critically ill trauma patients reduces ventilator days and hospital length of stay. J Trauma. 2008;65(3):517 526. 10.1097/TA.0b013e318181b8f6) 18784563

[3] Degrado JR Anger KE Szumita PM Pierce CD Massaro AF . Evaluation of a local ICU sedation guideline on goal-directed administration of sedatives and analgesics. J Pain Res. 2011;4:127 134. 10.2147/JPR.S18161) 21647216 PMC3100227

[4] Faust AC Rajan P Sheperd LA Alvarez CA McCorstin P Doebele RL . Impact of an analgesia-based sedation protocol on mechanically ventilated patients in a medical intensive care unit. Anesth Analg. 2016;123(4):903 909. 10.1213/ANE.0000000000001393) 27644010 PMC5237378

[5] Skrobik Y Chanques G . The pain, agitation, and delirium practice guidelines for adult critically ill patients: a post-publication perspective. Ann Intensive Care. 2013;3(1):9. 10.1186/2110-5820-3-9) PMC3622614 23547921

[6] Sneyers B Laterre PF Perreault MM Wouters D Spinewine A . Current practices and barriers impairing physicians’ and nurses’ adherence to analgo-sedation recommendations in the intensive care unit: a national survey. Crit Care. 2014;18(6):655. 10.1186/s13054-014-0655-1) PMC4324789 25475212

[7] McLean MJ Gabapentin. Epilepsia. 1995;36(suppl 2):S73 S86. 10.1111/j.1528-1157.1995.tb06001.x) 8784216

[8] Gee NS Brown JP Dissanayake VU Offord J Thurlow R Woodruff GN . The novel anticonvulsant drug, gabapentin (Neurontin), binds to the α2δ subunit of a calcium channel. J Biol Chem. 1996;271(10):5768 5776. 10.1074/jbc.271.10.5768) 8621444

[9] Barr J Donner A . Optimal intravenous dosing strategies for sedatives and analgesics in the intensive care unit. Crit Care Clin. 1995;11(4):827 847. 10.1016/S0749-0704(18)30041-1) 8535981

[10] Jacobi J Fraser GL Coursin DB , et al. Clinical practice guidelines for the sustained use of sedatives and analgesics in the critically ill adult. Crit Care Med. 2002;30(1):119 141. 10.1097/00003246-200201000-00020) 11902253

[11] Ramsay MA Savege TM Simpson BR Goodwin R . Controlled sedation with alphaxalone-alphadolone. Br Med J. 1974;2(5920):656 659. 10.1136/bmj.2.5920.656) 4835444 PMC1613102

[12] Payen JF Bru O Bosson JL , et al. Assessing pain in critically ill sedated patients by using a behavioral pain scale. Crit Care Med. 2001;29(12):2258 2263. 10.1097/00003246-200112000-00004) 11801819

[13] MacIntyre NR . Evidence-based guidelines for weaning and discontinuing ventilatory support: a collective task force facilitated by the American College of Chest Physicians; the American Association for Respiratory Care; and the American College of Critical Care Medicine. Chest. 2001;120(suppl 6):375S 395S. 10.1378/chest.120.6_suppl.375s) 11742959

[14] Fadaii A Amini SS Bagheri B Taherkhanchi B . Assessment of rapid shallow breathing index as a predictor for weaning in respiratory care unit. Tanaffos. 2012;11(3):28 31. 25191425 PMC4153203

[15] Salarian S Khosravi R Khanbabaei G Bagheri B . Impact of oral clonidine on duration of opioid and benzodiazepine use in mechanically ventilated children: a randomized, double-blind, placebo-controlled study. Iran J Pharm Res. 2019;18(4):2157 2162. 10.22037/ijpr.2019.14862.12705) 32184880 PMC7059056

[16] Salarian S Mirrahimi B Bagheri B . Rate of self-extubation in pediatric intensive care following administration of fentanyl, midazolam and midazolam-fentanyl combination: a comparative study. Int J Pediatr. 2018;6(1):6971 6976.

[17] Pandey CK Bose N Garg G , et al. Gabapentin for the treatment of pain in Guillain-Barré syndrome: a double-blinded, placebo-controlled, crossover study. Anesth Analg. 2002;95(6):1719 23, table of contents. 10.1097/00000539-200212000-00046) 12456446

[18] Pandey CK Raza M Tripathi M Navkar DV Kumar A Singh UK . The comparative evaluation of gabapentin and carbamazepine for pain management in Guillain-Barré syndrome patients in the intensive care unit. Anesth Analg. 2005;101(1):220 5, table of contents. 10.1213/01.ANE.0000152186.89020.36) 15976235

[19] Hosokawa R Ito S Hirokawa J Oshima Y Yokoyama T . Effectiveness of preanesthetic administration of gabapentin on sedative action during intravenous sedation with propofol. J Anesth. 2018;32(6):813 821. 10.1007/s00540-018-2559-8) 30238330

[20] Bharti N Bala I Narayan V Singh G . Effect of gabapentin pretreatment on propofol consumption, hemodynamic variables, and postoperative pain relief in breast cancer surgery. Acta Anaesthesiol Taiwan. 2013;51(1):10 13. 10.1016/j.aat.2013.03.009) 23711599

[21] Rusy LM Hainsworth KR Nelson TJ , et al. Gabapentin use in pediatric spinal fusion patients: a randomized, double-blind, controlled trial. Anesth Analg. 2010;110(5):1393 1398. 10.1213/ANE.0b013e3181d41dc2) 20418301

[22] Mayell A Srinivasan I Campbell F Peliowski A . Analgesic effects of gabapentin after scoliosis surgery in children: a randomized controlled trial. Paediatr Anaesth. 2014;24(12):1239 1244. 10.1111/pan.12524) 25230144

[23] Haney AL Garner SS Cox TH . Gabapentin therapy for pain and irritability in a neurologically impaired infant. Pharmacotherapy. 2009;29(8):997 1001. 10.1592/phco.29.8.997) 19637954

[24] Taylor CP Mechanisms of analgesia by gabapentin and pregabalin – calcium channel α2-δ [Cavα2-δ] ligands. Pain. 2009;142(1-2):13 16. 10.1016/j.pain.2008.11.019) 19128880

[25] Patel R Dickenson AH . Mechanisms of the gabapentinoids and α-2 δ-1 calcium channel subunit in neuropathic pain. Pharmacol Res Perspect. 2016;4(2):e00205. 10.1002/prp2.205) PMC4804325 27069626

[26] Morrell MJ McLean MJ Willmore LJ , et al. Efficacy of gabapentin as adjunctive therapy in a large, multicenter study. The Steps Study Group. Seizure. 2000;9(4):241 248. 10.1053/seiz.2000.0407) 10880282

[27] McLean MJ Morrell MJ Willmore LJ , et al. Safety and tolerability of gabapentin as adjunctive therapy in a large, multicenter study. Epilepsia. 1999;40(7):965 972. 10.1111/j.1528-1157.1999.tb00804.x) 10403221

[28] Devlin JW Skrobik Y Gélinas C , et al. Clinical practice guidelines for the prevention and management of pain, agitation/sedation, delirium, immobility, and sleep disruption in adult patients in the ICU. Crit Care Med. 2018;46(9):e825 e873. 10.1097/CCM.0000000000003299) 30113379

